# How Well Can We Assess Our Ability to Understand Others’ Feelings? Beliefs About Taking Others’ Perspectives and Actual Understanding of Others’ Emotions

**DOI:** 10.3389/fpsyg.2019.02475

**Published:** 2019-11-11

**Authors:** Jacob Israelashvili, Disa Sauter, Agneta Fischer

**Affiliations:** Department of Psychology, University of Amsterdam, Amsterdam, Netherlands

**Keywords:** emotion recognition, empathy, perspective taking, subjective beliefs, accuracy

## Abstract

People vary in their beliefs about their tendency to engage in perspective taking and to understand other’s feelings. Often, however, those beliefs are suggested to be poor indicators of actual skills and thus provide an inaccurate reflection of performance. Few studies, however, have examined whether people’s beliefs accurately predict their performance on emotion recognition tasks using dynamic or spontaneous emotional expressions. We report six studies (*N* ranges from 186 to 315; *N*_total_ = 1,347) testing whether individuals’ report of their engagement in perspective taking, as measured by the Interpersonal Reactivity Index (IRI; Davis, 1983), is associated with accurate emotion recognition. In Studies 1–3, emotion recognition performance was assessed using three standard tests of nonverbal emotion recognition. To provide a more naturalistic test, we then assessed performance with a new emotion recognition test in Studies 4–6, using videos of real targets that share their emotional experiences. Participants’ multi-scalar ratings of the targets’ emotions were compared with the targets’ own emotion ratings. Across all studies, we found a modest, yet significant positive relationship: people who believe that they take the other’s perspective also perform better in tests of emotion recognition (*r* = 0.20, *p* < 0.001). Beliefs about taking others’ perspective thus reflect interpersonal reality, but only partially.

## Introduction

*“The only true discovery, would not be to visit strange lands but to possess other eyes, to behold the universe through the eyes of another”*.

(Marcel Proust, 1922)

Attempts to understand others by “possessing their eyes” or “stepping into their shoes” are commonly considered an essential component of empathy (e.g., Davis, 1983; Preston and de Waal, 2002). Taking another’s perspective is typically deemed foundational for understanding others’ emotions (e.g., Batson et al., 2007; Erle and Topolinski, 2017). Previous research, however, has used different definitions and measures of perspective taking (for relevant discussions see: Keysers and Gazzola, 2014; Olderbak and Wilhelm, 2017; Hall and Schwartz, 2019; Murphy and Lilienfeld, 2019). One pertinent distinction is whether perspective taking is measured by asking people about their beliefs about their engagement in perspective taking, or by measuring skills that are assumed to be the result of the ability to take another’s perspective. The question that then arises is whether people’s beliefs reflect their actual skills. One reason why beliefs might mismatch skills is that people base their beliefs on subjective evaluation criteria (i.e., self-report) whereas actual skills are based on objective evaluation criteria (i.e., the actual performance). Subjective evaluation is likely to be biased because people show various positive biases when reporting on their own competence, dispositions, or habits; we do not know ourselves well, because we block unwanted feelings and thoughts (e.g., Wilson and Dunn, 2004). Even when we know ourselves, self-reports are biased by factors such as social desirability (Sedikides et al., 2003). On the other hand, in some domains research has shown that subjective measures can be valid and comparable with objective indicators, for example, in the case of well-being (e.g., Sandvik et al., 2009).

In the current paper, we test the relation between subjective beliefs and actual performance in perspective taking. Specifically, we examine the relationship between participants’ beliefs about their own propensity to take another’s perspective and a wide range of different recognition tasks. These recognition tasks range from tests using static pictures of actors, as have often been utilized in the existing literature, to tests including dynamic posed stimuli, and novel tests showing videos of targets sharing real-life emotional experiences.

## Perspective Taking

In the literature, definitions of Perspective Taking (PT) highlight the *propensity* to engage in perspective taking or the *ability* to accurately understand the inner states of others (see Keysers and Gazzola, 2014). For example, Davis (1983) refers to perspective taking as “the tendency to spontaneously adopt the psychological point of view of others in everyday life”; whereas Chrysikou and Thompson (2016) refer to perspective taking as “the ability to shift to another’s emotional perspective” (Chrysikou and Thompson, 2016). When perspective taking is operationalized, many studies use self-reports on perspective taking as an indicator of actual perspective taking ability (for reviews see Hall and Schwartz, 2019; Murphy and Lilienfeld, 2019).

There are several reasons why taking another person’s perspective may be associated with enhanced interpersonal accuracy. First, shifting attention toward others may increase the richness with which perceivers represent other’s states (Zaki, 2014). Second, perspective taking may lead perceivers to focus on expressive cues that communicate information about the feelings of others (e.g., eye region; Cowan et al., 2014). Third, perspective taking can reduce the reliance on known sources of error (e.g., self-projection; Zhang and Epley, 2009; Yaniv and Choshen-Hillel, 2012).

Several meta-analyses have been conducted to examine whether self-reported empathy accurately predicts interpersonal accuracy, including studies with different types of stimuli and different types of judgments (e.g., Davis and Kraus, 1997; Hall et al., 2009). Davis and Kraus (1997) examined a range of personality measures in their meta-analysis, but did not find dispositional empathy to be a significant predictor of interpersonal accuracy. One reason for the lack of an effect may be that they were unable to generate an accurate estimation of the relationship because many old studies reported imprecise statistical data (i.e., only values of *p* but not *r* coefficients)^1^. Moreover, while there is a clear distinction between different facets of empathy (e.g., perspective taking vs. empathic concern vs. personal distress; see discussions by Davis, 1983; Israelashvili and Karniol, 2018), many of the earlier studies in the field averaged all different facets of empathy into a global empathy score.

Recently, Murphy and Lilienfeld (2019) meta-analyzed more recent tests regarding the relation between self-reported perspective taking (i.e., IRI) and different cognitive-behavioral empathy tests. Their analysis showed that only 1% of the variance was explained by self-reported cognitive empathy, and the authors raise concerns with using self-reported empathy as a valid predictor for actual performance. Critically, however, their meta-analysis also showed substantial heterogeneity across findings. The relation between beliefs about engagement in perspective taking and actual performance ranged from a small negative effect (*r* = −0.16) to a strong positive effect (*r* = 0.48). Thus, although their meta-analysis provided a global mean estimation of the beliefs-performance relation (*r* = 0.10; equivalent to 1% explained variance), the high heterogeneity (*I*^2^ = 63.17; for interpretation see Higgins et al., 2003) of effects across the meta-analyzed studies violated the null hypothesis that all these effects evaluate the same relation, and consequently, lower the confidence in the averaged effect estimation. One explanation for the heterogeneity of effect sizes in this meta-analysis (see Murphy and Lilienfeld, 2019) may be the heterogeneity of stimuli used in the different tests [i.e., Reading the Mind in the Eyes test (RMET, Baron-Cohen et al., 2001); Profile of Nonverbal Sensitivity (PONS test, Rosenthal et al., 1977); Diagnostic Analysis of Nonverbal Accuracy (DANVA, Nowicki and Duke, 1994)], including static pictures of only the eye region of a single face to pictures of complex interpersonal social situations.

**Figure 1:**
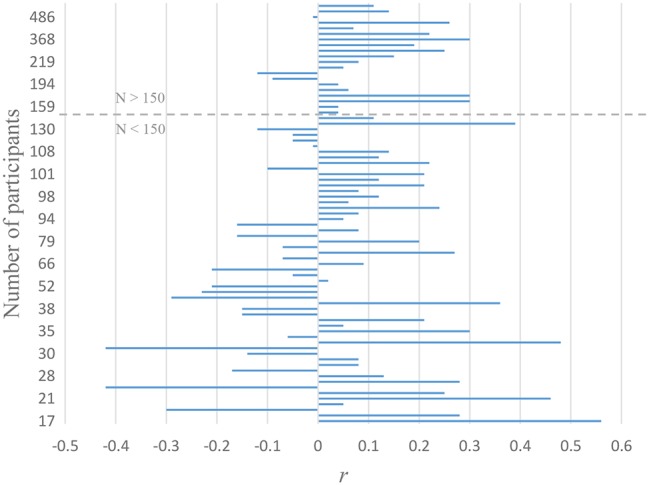
Display of effect sizes (i.e., magnitudes of correlations) for the relationship observed between self-reported perspective taking and emotion recognition tests, as observed in previous studies sorted by sample sizes. Note: This visualization was made using data from Murphy and Lilienfeld (2019) (Supplemental Table 1).

There are other reasons why a correlation between engagement in perspective taking and accurate emotion recognition may be inconsistent. Perspective taking is often a cognitively demanding task that requires time, motivation, and attentional resources to execute (Epley et al., 2004). One implication of engagement in perspective taking could thus be that participants pay less (rather than more) attention to others because they concentrate on their own egocentric experiences (e.g., Epley et al., 2004), which may lead to less accurate emotion recognition (e.g., Eyal et al., 2018). Finally, it is noteworthy that most of the studies that reported a positive relationship between beliefs that one takes others’ perspectives and the ability to recognize others’ emotions had a relatively large number of participants (see Figure 1). This observation may suggest that some of the variability in conclusions reported in the literature may be due to differences in power, with the more highly powered studies indicating a positive (albeit small) relation between propensity and ability to take others’ perspective and understand their emotions. Indeed, a power analysis (using G-power) indicates that to detect small to medium correlation (*r* = 0.2, one-tailed) with the standard criteria (*α* = 0.05, 1 − *β* = 0.80) would require 150 participants. Thus, some previous studies may have been underpowered for detecting the relation if the effect size is relatively small.

## The Present Research

The current study sought to examine the relation between perspective taking, as one aspect of empathy, and the performance on emotion recognition tests. Many theorists have claimed that perspective taking ability and emotion recognition should be closely related (e.g., Preston and de Waal, 2002; Epley et al., 2004; Erle and Topolinski, 2017), but as discussed, the evidence for this claim is limited. The goal of the current research was to examine how robust the relation is between individuals’ beliefs about their tendency to engage in perspective taking and their actual performance on verbal and nonverbal tests of emotional accuracy. Based on the heterogeneity of findings reported in the literature described above, we did not make any prediction *a priori*. Instead, we used data from six different studies conducted in our lab during the past 2 years (2017–2019), which all included self-reported beliefs about participants’ engagement in perspective taking and at least one emotion recognition test (see review in Table 1). In these studies, beliefs about engagement in perspective taking, as measured with the IRI, were collected for exploratory reasons, but were not discussed in the publications resulting from that work because they were not directly relevant to the primary research question. By combining different samples and instruments of emotion recognition, we were interested in assessing the generalizability of the beliefs-ability relation and generating a reliable estimation of effect size. Although previous meta-analyses have generated an estimation of the effect (*r =* 0.10), their reliance on static posted expressions may have biased their estimation (Hall et al., 2009; Murphy and Lilienfeld, 2019). In particular, when the recognition task is easy or boring it could produce limited variability in assessment of performance (due to ceiling/floor effects, respectively), which in turn can reduce its correlation with other constructs (e.g., perspective taking). In the current study, we focus on recognition tests with relatively high ecological validity. Rather than relying only on recognition of posed facial expressions by actors and defining accuracy as agreement with theoretically posited configurations, we used dynamic expressions and defined accuracy as perceivers’ agreement with the judgments of the individuals who experienced the actual emotions shared in the videos. To assess individuals’ beliefs about their own perspective taking, we used the Perspective Taking (PT) subscale of the Interpersonal Reactivity Index (IRI; Davis, 1983), as this is the most widely used measure of empathic tendencies (Hall and Schwartz, 2019). Individuals’ self-report of PT as measured by the IRI (Davis, 1983) has been found to constitute a significant predictor of whether perceivers focus on expressive cues that communicate information about the feelings of others (e.g., eye region; Cowan et al., 2014) and the extent to which perceivers show physiological arousal in response to others’ emotional states (e.g., van der Graaff et al., 2016).

**Table 1:** Description of the emotion recognition tasks used in Studies 1–6 and their correlation with perspective taking.

Study	Test#	Task (emotional cue)	Stimuli	Emotional expression	*N*	%Females	ES	SE
Study 1	1	AERT (face)	Static (picture)	Posed	245	0.53	0.20^**^	0.06
Study 1	2	RMET (eyes)	Static (picture)	Posed	245	0.53	0.16^*^	0.06
Study 1	4	GERT (face, posture and voice)	Dynamic (videos)	Posed	245	0.53	0.24^***^	0.06
Study 2	3	RMET (eyes)	Static (picture)	Posed	186	0.47	0.20^**^	0.07
Study 3	5	GERT (face, posture and voice)	Dynamic (videos)	Posed	315	0.39	0.17^*^	0.06
Study 4	6	Novel task (face, voice and words)	Dynamic (videos set #1)	Spontaneous	207	0.49	0.09	0.07
Study 5	7	Novel task (face, voice and words)	Dynamic (videos set #2)	Spontaneous	207	0.41	0.26^***^	0.07
Study 6	8	Novel task (face, voice and words)	Dynamic (videos set #2)	Spontaneous	187	0.40	0.36^***^	0.08

*AERT, Amsterdam Emotion Recognition Test; GERT, Geneva Emotion Recognition Test; RMET, Reading the Mind in the Eyes Test; ES, effect size (based on Spearman’s correlation); SE, standard error*.

* *p < 0.05*;

** *p < 0.01*;

*** *p < 0.001*.

To assess accurate emotion recognition, we used three standard tests of nonverbal emotion recognition in Studies 1–3: the Reading the Mind in the Eyes Test (RMET; Baron-Cohen et al., 2001), the Amsterdam Emotion Recognition Task (AERT; Van Der Schalk et al., 2011; Israelashvili et al., 2019), and the Geneva Emotion Recognition Test (GERT; Schlegel et al., 2014). In Studies 4–6, emotion recognition was assessed using a novel paradigm with dynamic videos of targets who share their genuine emotional experiences in a 2-min video. Participants (perceivers) were asked to identify the emotions that the targets expressed in video clips. Using the targets’ independent multi-scalar ratings of their own emotions, we calculated emotion recognition accuracy, operationalized as the similarity between each target’s and perceiver’s emotion ratings (see section “Methods”). With the different instruments and samples included in this analysis, we sought to provide a robust test of the relation between beliefs about taking others’ perspectives (i.e., PT) and performance across different emotion recognition tasks.

## Methods

### Participants and Procedure

Participants in Studies 1–6 were 1,347 US citizens (*Study 1*: *N* = 245, 53% females*, M*_age_ = 37, SD_age_ = 12; *Study 2*: *N* = 186, 46% females, *M*_age_ = 37, SD_age_ = 12; *Study 3*: *N* = 315, 39% females; *Study 4*: *N* = 207, 49% females, *M*_age_ = 37, SD_age_ = 11; *Study 5: N* = 201, 60% females, *M*_age_ = 38, SD_age_ = 13; *Study 6: N* = 187, 47% females, *M*_age_ = 38, SD_age_ = 13)^2^, who were recruited *via* Amazon Mechanical Turk (Mturk). We restricted the MTurk sample to individuals with a high reputation (i.e., above 95% approved ratings; see Peer et al., 2014). In addition, we allowed only individuals in the USA to take part because American participants have their worker ID associated with their Social Security Number, which reduces the risk of people taking the same survey multiple times with different identities. The description was identical for all studies: “*View people in various situations and rate their emotions*.” Informed consent was obtained from all participants, and the procedure was approved by the Ethics Committee of the University of Amsterdam. Participants completed the IRI questionnaire and one or more emotion recognition tests as part of a more extensive test session, which addressed several different research questions (i.e., whether interpersonal accuracy relates to individual differences in emotion differentiation ability, relates to the feeling of similarity in experience, or relates to the feeling of concern and distress). Here we only present results on the correlation between PT and accurate emotion recognition. The results for the other measures were discussed in the relevant papers (see Israelashvili et al., 2019; in press). We did not exclude participants from our analyses, except for participants in Study 2, 5, and 6 who failed to answer attention check question correctly (see Israelashvili et al., in press). The number of participants who performed in any recognition test below chance level was minimal (2%), and the pattern of results reported in the paper is identical whether those individuals were excluded or not. A sensitivity analysis conducted in G-power suggested that with the standard criteria (*α* = 0.05), the analysis of correlations has a power of 0.80 to detect a small to medium effect (*r* = 0.2), with each of the samples included in the analysis.

### Measures

#### The Interpersonal Reactivity Index (Studies 1–6)

The IRI is a 28-item self-report scale, tapping four components of dispositional empathy, of which two represent cognitive components (Perspective Taking, Fantasy), and two represent affective components (Empathic Concern, Personal Distress) (Davis, 1983, 1994). Here we focus only on the seven-item subscale of *Perspective Taking* (PT) – a subscale measuring the tendency to imagine other people’s points of view (e.g., “I sometimes try to understand my friends better by imagining how things look from their perspective”). Participants rate their agreement with each item on a five-point Likert scale, ranging from 1 = *does not describe me well*, to 5 = *describes me very well*. Cronbach’s α reliabilities of the PT subscale in Study 1 were: PT = 0.*90*, in Study 2: PT = 0.82, in Study 3: PT = 0.88, in Study 4: PT = 0.88, in Study 5: PT = 0.83, and in Study 6: PT = 0.84. The means (and standard deviations) of the PT subscale in Study 1 were: PT = 3.69 (SD = 0.94), in Study 2: PT = 3.51 (SD = 0.78), in Study 3: PT = 3.42 (SD = 0.73), in Study 4: PT = 3.52 (SD = 0.90), in Study 5: PT = 3.29 (SD = 0.86), and in Study 6: PT = 3.53 (SD = 0.90).

#### Amsterdam Emotion Recognition Test (Study 1)

The AERT comprises 24 photos of four models (two males and two females) who display six negative emotions (anger, fear, sadness, embarrassment, contempt, and disgust) with low intensity (for more details, see Israelashvili et al., 2019). Participants were asked to label the emotion they saw on the face by selecting one of six emotion labels, or “I do not know.” Responses were scored as correct (1) or incorrect (0). Accurate emotion recognition was operationalized by calculating the percentage of correct answers across the 24 pictures. This test was used only in Study 1 (reliability Cronbach’s *α* = 0.70), and the overall recognition rate was 62% (SD = 16%).

#### Reading the Mind in the Eyes (Studies 1 and 2)

The RMET comprises 36 photos depicting the eye region of 36 White individuals (Baron-Cohen et al., 2001). Participants are asked to identify the emotional state of a target person, whose eye region is shown in a photograph, by choosing one out of four words that each represents an emotional state (e.g., serious, ashamed, alarmed, or bewildered). Responses are scored as correct (1) or incorrect (0); the RMET score is calculated by summing the correct answers. The performance was determined by calculating the percentage of correct responses. This test was used only in Studies 1 and 2. The reliability (Cronbach’s α) of the test in Study 1 was = 0.8*4*, and in Study 2 = 0.88. The average recognition in Study 1 was 73% (SD = 16%) and in Study 2 66% (SD = 20%).

#### Geneva Emotion Recognition Test (Studies 1 and 3)

We used the short version of the Geneva Emotion Recognition Test (Schlegel et al., 2014). The test consists of 42 short video clips with sound (duration 1–3 s), in which 10 professional White actors (five male and five female) express 14 different positive and negative emotions: joy, amusement, pride, pleasure, relief, interest, surprise, anger, fear, despair, irritation, anxiety, sadness, and disgust. In each video clip, the actor is visible from their upper torso upward (conveying facial and postural/gestural emotional cues) and pronounces a sentence made up of syllables without semantic meaning. After each clip, participants were asked to choose which one out of the 14 emotions best describes the emotion the actor intended to express. Responses were scored as correct (1) or incorrect (0). Similar to RMET and AERT, the final GERT score was calculated as the percentage of accurate recognitions ranging from 0 to 100%. This test was also used only in Studies 1 and 3 (reliability in Study 1: Cronbach’s *α* = 0.78, and in Study 3: Cronbach’s *α* = 0.74), and the average recognition in Study 1 was 55% (SD = 15%) and in Study 3 was 58% (SD = 14%).

#### Accurate Emotion Recognition (Novel Task; Studies 4–6)

To measure emotion recognition in a way that better approximates real life, we developed a new measure of perceivers’ ability to accurately recognize a target’s emotional state from video clips. In Studies 4–6, participants watched four video clips in a random order. All videos were between 2 and 3 min long, and each consisted of an English-speaking female in her early 20s freely describing a genuine emotional autobiographical experience. The targets were asked to share an actual emotional experience from their own life that they felt comfortable sharing. The topics of the four videos used in Study 4 were: (1) fear of breakup, (2) signs of a partner cheating, (3) reverse culture shock, and (4) fighting with a parent. The topics of the four videos used in Studies 5–6 were: (1) experience of a parent being ill, (2) a divorced father in a new relationship, (3) emotional distance from family, and (4) problems with an internship. After sharing the emotional experience, we asked the targets to watch their own video and to rate the emotions that they had felt in that video. Each target watched her video and then rated the intensity with which she experienced each of 10 emotions (anger, rage, disappointment, fear, sadness, worry, confusion, surprise, embarrassment, and guilt). Answers were given on a seven-point Likert scale, ranging from (0) *not at all* to (6) *very much*. In each study, participants were asked to watch the videos and to rate the intensity with which they thought the target experienced each of 10 emotions using the same list of emotions as that used by the targets. Accuracy was calculated based on the absolute difference between participants’ ratings and the target’s own ratings, across each one of the 10 emotion rating scales (larger absolute differences indicate lower accuracy; for a similar approach see: Zhou et al., 2017; Eyal et al., 2018). We used the average accuracy score across all targets as the unit of analysis, consistent with previous research on empathic accuracy and emotion recognition (e.g., Zaki et al., 2008; Eckland et al., 2018; Mackes et al., 2018), and consistent with the average measure used for other recognition tasks (AERT, RMET, GERT). Finally, to simplify the interpretation of this index, the average absolute difference was reversed (−1* average absolute difference), such that a higher score in this index reflects better accuracy. The average absolute distance in Study 4 was 16.10 (SD = 4.64), in Study 5 was 18.91 (SD = 5.19), and in Study 6 was 18.43 (SD = 5.47).

## Results

### Meta-Analysis

**Figure 2:**
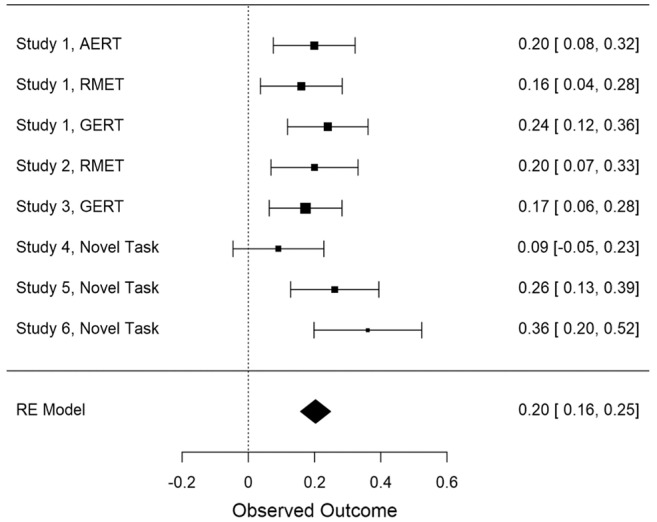
Forest plot of the effect size of the relation between self-reported Perspective Taking and accuracy estimated using the random effects (RE) model. For each test of the relation, the size of the box represents the mean effect size estimate, which indicates the weight of that study in the meta-analysis. Numerical values in each row indicate the mean and 95% confidence interval of effect size estimates in bootstrapping analyses. AERT, Amsterdam Emotion Recognition Task; GERT, Geneva Emotion Recognition Test; RMET, Reading the Mind in the Eyes Test.

To try to identify a robust pattern of relations between individuals’ self-report of perspective taking and their actual performance on recognition tasks, we conducted a meta-analysis. Since the variables under study were not normally distributed (Shapiro-Wilk >0.96, *p* < 0.001 across all studies), we used Spearman’s correlation coefficient as a measure of the relation (though it should be noted that we obtained the same results with Pearson correlations). Because Studies 1–6 used several tests of emotion recognition, we conducted a random-effects meta-analysis, using the JASP 0.9.2 software (JASP Team, 2018). The meta-analysis utilized eight different tests of the relation (Spearman’s correlation) between self-reported perspective taking and emotion recognition, based on the tasks reported in Table 1. The meta-analysis yielded a positive relationship estimated as 0.20, 95% CI (0.15, 0.24), *Z* = 8.782, *p* < 0.001 (see Figure 2). In addition, we calculated the heterogeneity of the observed effect sizes to test whether our estimate of the average effect (*r* = 0.2) can be generalized. Findings indicated that random sampling differences alone can produce the small variance of the observed effect, *Q* = 8.03, *I*^2^ = 0.07, df = 7, *p* = 0.33, and thus, that the estimate can be generalized across measures and studies included in the analysis. This finding indicates that individual differences in engagement in perspective taking are positively related to the performance on experimental tasks of emotion recognition.

## Discussion

In a series of six studies, using classic emotion recognition tests with posed static and dynamic stimuli, as well as spontaneous dynamic stimuli, we examined whether individuals’ *beliefs* about their tendency to engage in perspective taking aligns with their actual *performance*. The result from a meta-analysis of our findings indicates that individuals high in self-reported perspective taking also perform better on tests of emotion recognition.

The findings reported here are comparable with findings obtained in previous meta-analyses on the relation between empathy and interpersonal skills (e.g., Hall et al., 2009; Murphy and Lilienfeld, 2019), which show a similar small positive relation between PT and emotion recognition. The focus of previous studies was limited, however, to recognition of static stimuli (i.e., pictures). To our knowledge, the current research is the first to demonstrate a positive relation using dynamic emotion recognition tests. In particular, the current research utilizes both spontaneous and posed dynamic expressions of emotions with verbal as well as nonverbal emotional cues, and thus, arguably has high ecological validity. Thus, the findings reported here and in previous research point to belief about everyday engagement in perspective taking partially reflecting interpersonal reality.

It is worth noting that the observed effect was robust but relatively small in magnitude. Cohen’s convention guideline (Cohen, 1992) to interpret the correlation coefficients argues that *r* = 0.10 represents a small effect size, whereas *r =* 0.30 represents a medium effect size. For interpretations of meta-analysis findings, Hemphill (2003) has argued that relationship of *r =* 0.20 should be interpreted as medium effect size, since only one-third of all correlation coefficients show values higher than *r =* 0.30 according to an analysis of 380 meta-analyses findings in psychology. The observed correlation [*r* = 0.20, 95%CI (0.16, 0.25)] thus indicates a small to medium effect size.

The magnitude of the relation should be interpreted within the range of correlations relevant to the field. A recent meta-analysis of more than 100 samples probing different performance tests of emotion recognition ability showed that performance on different tasks correlates only modestly with one another (*r* = 0.29; Schlegel et al., 2017; Table 5), even though they are believed to assess the same underlying construct (emotion recognition ability). Moreover, performance tests are poor at differentiating between individuals across the theoretical continuum of emotion recognition ability. For example, people diagnosed with autism and a group of healthy matched-IQ controls differ on emotion recognition test only to modest levels (equivalent to *r* values between 0.17 and 0.27, or 3–8% explained variance; calculations based on the results reported in Jones et al., 2010; Sucksmith et al., 2013). Accordingly, we believe that even an effect that is modest in size (like the *r* = 0.20 in the current findings), in the current field of research, might capture meaningful individual differences.

Importantly, the present meta-analysis is based on emotion recognition of a wide range of stimuli, using minimal emotional cues involving the static expression of only the eyes region as well as multimodal verbal and nonverbal expressions of emotions in dynamic videos. Yet, the heterogeneity of the experimental setting was limited to expressions of emotions in a relatively short time period presented in pictures or videos. It is conceivable that the relationship between individuals’ beliefs about their perspective taking propensity and their actual ability to infer how others feel may potentially be stronger in daily life situations, because when people state their everyday engagement in perspective taking, they likely refer to their typical behavior. This typical behavior naturally happens in a social environment that involves others they care about and, consequently, whose perspectives and feelings they care to understand their perspectives and feelings. For example, people wish to understand the emotions of a beloved or influential other because it is relevant for their own life. Thus, what drives people to engage in perspective taking is often related to relational motives and in particular, feeling empathic concern for others (Hodges et al., 2018; see also, Zaki, 2014; Israelashvili and Karniol, 2018; Batson, 2019). In an experimental environment, the content of others’ emotions has limited relevance to the perceiver’s life, and thus, this context is often characterized by low engagement.

Furthermore, from a methodological perspective, studies of emotion recognition measure how well people perform when asked to perform to the best of their ability (i.e., Maximal behavior) on standardized tests with veridical answers. However, assessment of accuracy based on *Maximal behavior* during test sessions and assessment of beliefs about understanding others based on *typical behavior* relies on different measurement levels (maximal vs. typical behavior, respectively; see discussions by Cronbach, 1949; Olderbak and Wilhelm, 2017). This mismatch of measurement levels may result in an underestimation of the examined relation compared to when similar levels of measurement are used. In sum, we believe that current research findings of a small to medium effect size might show a smaller relation than the actual size of the relation in real-life (naturalistic situations). Thus, we suggest that the observed positive relation is meaningful.

Unfortunately it is not possible to use participants’ self-reported tendencies to engage in perspective taking as a proxy for their actual abilities. Beliefs are subjective features accessed *via* self-report, whereas skills are objective features that require behavioral assessment of actual performance in relevant tasks (see also Davis and Kraus, 1997; Murphy and Lilienfeld, 2019). Given this difference, researchers and clinicians should regard them as *complementary* sources of information. After all, successful social functioning requires both the motivation and the ability to engage in perspective taking and accurately recognize others’ feelings (e.g., Carpenter et al., 2016).

One limitation of the current analysis is the use of a correlational design, which does not allow us to address the question of causality. It may be that accurate recognition of emotional cues triggers engagement in perspective taking (e.g., Frith and Frith, 2006), or vice versa. Furthermore, as mentioned above, the investigation of the research question in the current and previous studies was limited to tests with expression of emotions occurring in a relatively short amount of time. Future research is needed to examine the relation in a naturalistic setting.

## Conclusion

When the Marist Institute for Public Opinion asked a poll of 1,020 Americans what superpower they would most like to have, the ability to read the minds of others was mentioned as one of the two most desired qualities (together with traveling in time; Marist, 2011). This survey suggests that people are aware that their understanding of the thoughts and feelings of others often fail short of perfection. To understand other people better, some individuals tend to engage in spontaneous attempts to understand others’ minds by taking their perspective. Here we report the result of a series of studies that examines whether people’s self-reported propensity to take others’ perspectives accurately predict their performance on emotion recognition tasks. We found that individuals high in perspective taking also perform better across a broad range of different tasks of emotion recognition. Thus, beliefs about engaging in perspective taking partially reflect interpersonal reality.

## Data Availability Statement

The datasets generated for this study are available on request to the corresponding author.

## Ethics Statement

The studies involving human participants were reviewed and approved by the Ethics Committee of the University of Amsterdam. The patients/participants provided their written informed consent to participate in this study.

## Author Contributions

All authors listed have made a substantial, direct and intellectual contribution to the work, and approved it for publication.

### Conflict of Interest

The authors declare that the research was conducted in the absence of any commercial or financial relationships that could be construed as a potential conflict of interest.

## Footnotes

^1^As the authors note: “in many cases the findings were reported simply as not-significant and in those instances we assume *r =* 0.00” (Davis and Kraus, 1997, p. 156).

^2^We report in the main text the rounded values of age and gender. The age of the participants in Study 3 was not saved due to technical problem.

## References

[ref1] Baron-Cohen S. Wheelwright S. Hill J. Raste Y. Plumb I. (2001). The “reading the mind in the eyes” test revised version: a study with normal adults, and adults with asperger syndrome or high-functioning autism. J. Child Psychol. Psychiatry 42, 241–251. 10.1017/S0021963001006643 11280420

[ref2] Batson C. D. (2019). A scientific search for altruism. New York: Oxford University Press.

[ref3] Batson C. D. Eklund J. H. Chermok V. L. Hoyt J. L. Ortiz B. G. (2007). An additional antecedent of empathic concern: valuing the welfare of the person in need. J. Pers. Soc. Psychol. 93, 65–74. 10.1037/0022-3514.93.1.65, PMID: 17605589

[ref4] Carpenter J. M. Green M. C. Vacharkulksemsuk T. (2016). Beyond perspective-taking: mind-reading motivation. Motiv. Emot. 40, 358–374. 10.1007/s11031-016-9544-z

[ref5] Chrysikou E. G. Thompson W. J. (2016). Assessing cognitive and affective empathy through the interpersonal reactivity index: an argument against a two-factor model. Assessment 23, 769–777. 10.1177/1073191115599055, PMID: 26253573

[ref382] Cohen J. (1992). Statistical power analysis. Curr. Dir. Psychol. Sci. 1, 98–101.

[ref6] Cowan D. G. Vanman E. J. Nielsen M. (2014). Motivated empathy: the mechanics of the empathic gaze. Cognit. Emot. 28, 1522–1530. 10.1080/02699931.2014.890563, PMID: 24568562

[ref7] Cronbach L. J. (1949). Essentials ofpsychological testing. New York, NY: Harper & Brothers.

[ref8] Davis M. H. (1983). Measuring individual differences in empathy: evidence for a multidimensional approach. J. Pers. Soc. Psychol. 44, 113–126. 10.1037/0022-3514.44.1.113

[ref9] Davis M. (1994). Empathy: A social psychological approach. New York: Westview Press.

[ref10] Davis M. H. Kraus L. A. (1997). “Personality and empathic accuracy” in Empathic accuracy. ed. Ickes W. (New York: Guilford), 144–168.

[ref11] Eckland N. S. Leyro T. M. Mendes W. B. Thompson R. J. (2018). A multi-method investigation of the association between emotional clarity and empathy. Emotion 18, 638–645. 10.1037/emo0000377, PMID: 29172622

[ref13] Epley N. Keysar B. Van Boven L. Gilovich T. (2004). Perspective taking as egocentric anchoring and adjustment. J. Pers. Soc. Psychol. 87, 327–339. 10.1037/0022-3514.87.3.327, PMID: 15382983

[ref14] Erle T. M. Topolinski S. (2017). The grounded nature of psychological perspective-taking. J. Pers. Soc. Psychol. 112, 683–695. 10.1037/pspa0000081, PMID: 28253002

[ref15] Eyal T. Steffel M. Epley N. (2018). Perspective mistaking: accurately understanding the mind of another requires getting perspective, not taking perspective. J. Pers. Soc. Psychol. 114, 547–571. 10.1037/pspa0000115, PMID: 29620401

[ref16] Frith C. D. Frith U. (2006). The neural basis of mentalizing. Neuron 50, 531–534. 10.1016/j.neuron.2006.05.001, PMID: 16701204

[ref21] Hall J. A. Andrzejewski S. A. Yopchick J. E. (2009). Psychosocial correlates of interpersonal sensitivity: a meta-analysis. J. Nonverbal Behav. 33, 149–180. 10.1007/s10919-009-0070-5

[ref22] Hall J. A. Schwartz R. (2019). Empathy present and future. J. Soc. Psychol. 159, 225–243. 29781776 10.1080/00224545.2018.1477442

[ref383] Hemphill J. F. (2003). Interpreting the magnitudes of correlation coefficients. Am. Psychol. 58, 78–79. 10.1037/0003-066x.58.1.78 12674822

[ref24] Hodges S. D. Denning K. R. Lieber S. (2018). Perspective taking: motivation and impediment to shared reality. Curr. Opin. Psychol. 23, 104–108. 10.1016/j.copsyc.2018.02.007, PMID: 29514123

[ref380] Higgins J. P. Thompson S. G. Deeks J. J. Altman D. G. (2003). Measuring inconsistency in meta-analyses. Br. med. J. 327, 557–560. 10.1136/bmj.327.7414.557, PMID: 12958120 PMC192859

[ref27] Israelashvili J. Karniol R. (2018). Testing alternative models of dispositional empathy: the affect-to-cognition (ACM) versus the cognition-to-affect (CAM) model. Personal. Individ. Differ. 121, 161–169. 10.1016/j.paid.2017.09.036

[ref28] Israelashvili J. Oosterwijk S. Sauter D. Fischer A. (2019). Knowing me, knowing you: emotion differentiation in oneself is associated with recognition of others’ emotions. Cognit. Emot. 1–11. PMID: 30734635 10.1080/02699931.2019.1577221

[ref280] Israelashvili J. Sauter D. Fischer A. (in press). Different faces of empathy: Feelings of similarity disrupt recognition of negative emotions. J. Exp. Soc. Psychol. 10.1016/j.jesp.2019.103912 PMC7001982 32127724

[ref29] JASP Team (2018). JASP (Version 0.9.2) [Computer software].

[ref30] Jones A. P. Happé F. G. Gilbert F. Burnett S. Viding E. (2010). Feeling, caring, knowing: different types of empathy deficit in boys with psychopathic tendencies and autism spectrum disorder. J. Child Psychol. Psychiatry 51, 1188–1197. 10.1111/j.1469-7610.2010.02280.x, PMID: 20633070 PMC3494975

[ref31] Keysers C. Gazzola V. (2014). Dissociating the ability and propensity for empathy. Trends Cogn. Sci. 18, 163–166. 10.1016/j.tics.2013.12.011, PMID: 24484764 PMC4560165

[ref32] Mackes N. K. Golm D. O’Daly O. G. Sarkar S. Sonuga-Barke E. J. Fairchild G.. (2018). Tracking emotions in the brain–revisiting the empathic accuracy task. NeuroImage 178, 677–686. 10.1016/j.neuroimage.2018.05.080, PMID: 29890323 PMC6057276

[ref340] Marcel P. (1922). A la recherche du temps perdu. (English translation: Remembrance of things past). New York: Random.

[ref34] Marist P. (2011). McClatchy-Marist poll for public opinion. Available at: http://maristpoll.marist.edu/28-holy-super-powers-batman-mind-reading-and-time-travel-top-list/#sthash.WtR3JYo8.dpbs (Accessed October 27, 2019).

[ref36] Murphy B. A. Lilienfeld S. O. (2019). Are self-report cognitive empathy ratings valid proxies for cognitive empathy ability? Negligible meta-analytic relations with behavioral task performance. Psychol. Assess. 31, 1062–1072. 10.1037/pas0000732, PMID: 31120296

[ref37] Nowicki S. Duke M. P. (1994). Individual differences in the nonverbal communication of affect: the diagnostic analysis of nonverbal accuracy scale. J. Nonverbal Behav. 18, 9–35. 10.1007/BF02169077

[ref38] Olderbak S. Wilhelm O. (2017). Emotion perception and empathy: an individual differences test of relations. Emotion 17, 1092–1106. 10.1037/emo0000308, PMID: 28358563

[ref39] Peer E. Vosgerau J. Acquisti A. (2014). Reputation as a sufficient condition for data quality on Amazon mechanical Turk. Behav. Res. Methods 46, 1023–1031. 10.3758/s13428-013-0434-y, PMID: 24356996

[ref40] Preston S. D. de Waal F. B. M. (2002). Empathy: its ultimate and proximate bases. Behav. Brain Sci. 25, 1–20. 10.1017/S0140525X02000018, PMID: 12625087

[ref381] Rosenthal R. Hall J. A. Archer D. DiMatteo M. R. Rogers P. L. (1977). “The PONS Test: Measuring sensitivity to nonverbal cues” in Advances in psychological assessment (San Francisco: Josser-Bass). PMID:

[ref42] Sandvik E. Diener E. Seidlitz L. (2009). “Subjective well-being: the convergence and stability of self-report and non-self-report measures” in Assessing well-being. eds. Diener E. D. (Dordrecht: Springer), 119–138.

[ref43] Schlegel K. Boone R. T. Hall J. A. (2017). Individual differences in interpersonal accuracy: a multi-level meta-analysis to assess whether judging other people is one skill or many. J. Nonverbal Behav. 41, 103–137. 10.1007/s10919-017-0249-0

[ref44] Schlegel K. Grandjean D. Scherer K. R. (2014). Introducing the Geneva emotion recognition test: an example of Rasch-based test development. Psychol. Assess. 26, 666–672. 10.1037/a0035246, PMID: 24295238

[ref45] Sedikides C. Gaertner L. Toguchi Y. (2003). Pancultural self-enhancement. J. Pers. Soc. Psychol. 84, 60–79. 10.1037/0022-3514.84.1.60, PMID: 12518971

[ref46] Sucksmith E. Allison C. Baron-Cohen S. Chakrabarti B. Hoekstra R. A. (2013). Empathy and emotion recognition in people with autism, first-degree relatives, and controls. Neuropsychologia 51, 98–105. 10.1016/j.neuropsychologia.2012.11.013, PMID: 23174401 PMC6345368

[ref47] van der Graaff J. Meeus W. de Wied M. van Boxtel A. van Lier P. A. C. Koot H. M. (2016). Motor, affective and cognitive empathy in adolescence: interrelations between facial electromyography and self-reported trait and state measures. Cognit. Emot. 30, 745–761. 10.1080/02699931.2015.1027665 25864486

[ref48] Van Der Schalk J. Hawk S. T. Fischer A. H. Doosje B. (2011). Moving faces, looking places: validation of the Amsterdam dynamic facial expression set (ADFES). Emotion 11, 907–920. 10.1037/a0023853, PMID: 21859206

[ref50] Wilson T. D. Dunn E. W. (2004). Self-knowledge: its limits, value, and potential for improvement. Annu. Rev. Psychol. 55, 493–518. 10.1146/annurev.psych.55.090902.141954, PMID: 14744224

[ref51] Yaniv I. Choshen-Hillel S. (2012). When guessing what another person would say is better than giving your own opinion: using perspective-taking to improve advice-taking. J. Exp. Soc. Psychol. 48, 1022–1028. 10.1016/j.jesp.2012.03.016

[ref52] Zaki J. (2014). Empathy: a motivated account. Psychol. Bull. 140, 1608–1647. 10.1037/a0037679, PMID: 25347133

[ref53] Zaki J. Bolger N. Ochsner K. (2008). It takes two: the interpersonal nature of empathic accuracy. Psychol. Sci. 19, 399–404. 10.1111/j.1467-9280.2008.02099.x, PMID: 18399894

[ref54] Zhang Y. Epley N. (2009). Self-centered social exchange: differential use of costs versus benefits in prosocial reciprocity. J. Pers. Soc. Psychol. 97, 796–810. 10.1037/a0016233, PMID: 19857002

[ref55] Zhou H. Majka E. A. Epley N. (2017). Inferring perspective versus getting perspective: underestimating the value of being in another person’s shoes. Psychol. Sci. 28, 482–493. 10.1177/0956797616687124, PMID: 28406380

