# Supplementary material for: How Well Can We Assess Our Ability to Understand Others’ Feelings? Beliefs About Taking Others’ Perspectives and Actual Understanding of Others’ Emotions
Source: Front Psychol. 2019 Nov 11;10:2475. doi: 10.3389/fpsyg.2019.02475 (PMC6882378; doi:10.3389/fpsyg.2019.02475)
Supplement: Supplementary file 1 [file Table_1.docx]

| Supplemental Table 1.  *Characteristics of Effect Sizes Included in the Meta-Analysis (non clinical = )* | | | | | | | |
| --- | --- | --- | --- | --- | --- | --- | --- |
|  |  |  |  |  |  |  |  |
| Study name | Subgroup (if any) | N | SR empathy dimension | Multisensory  CE test (Y/N) | CE test measure | SR measure | *r* |
| 1. Cavojova et al. (2011) | Girls | 101 | PT | yes | AMT | IRI PT | .21 |
| 1. Cavojova et al. (2011) | Boys | 96 | PT | yes | AMT | IRI PT | .24 |
| 1. Corbera et al. (2013) |  | 54 | PT | yes | BLERT | IRI PT | .02 |
| 1. Muller et al. (2010) |  | 30 | PT | no | Comic | IRI PT | -.17 |
| 1. Mullins-Nelson et al. (2006) | Men | 44 | PT | no | DANVA Faces | IRI PT | -.29 |
| 1. Gummelt (2014) ^a^ | Women | 101 | PT | no | DANVA faces | IRI PT | -.10 |
| 1. Gummelt (2014) ^a^ | Men | 128 | PT | no | DANVA faces | IRI PT | -.05 |
| 1. Mullins-Nelson et al. (2006) | Women | 130 | PT | no | DANVA Faces | IRI PT | -.05 |
| 1. Olderbak & Wilhelm (2017), Study 2 |  | 486 | PT | no | DANVA faces | IRI PT | -.01 |
| 1. Vachon & Lynam (2016) |  | 210 | PT | no | DANVA postures | IRI PT | -.12 |
| 1. Mullins-Nelson et al. (2006) | Men | 44 | PT | no | DANVA Voices | IRI PT | -.23 |
| 1. Mullins-Nelson et al. (2006) | Women | 130 | PT | no | DANVA Voices | IRI PT | -.12 |
| 1. Devlin et al. (2014) | Men | 52 | PT | yes | Emp Acc | IRI PT | -.21 |
| 1. Ickes et al. (1990) | Men | 38 | PT | yes | Emp Acc | IRI PT | -.15 |
| 1. Ickes et al. (1990) | Women | 38 | PT | yes | Emp Acc | IRI PT | -.15 |
| 1. Gleason et al. (2009) |  | 116 | PT | yes | Emp Acc | IRI PT | -.01 |
| 1. Laurent & Hodges (2009) |  | 194 | PT | yes | Emp Acc | IRI PT | .04 |
| 1. Brook & Kosson, (2013; also see Brook, 2011) |  | 100 | PT | yes | Emp Acc | IRI PT | .21 |
| 1. Devlin et al. (2014) | Women | 69 | PT | yes | Emp Acc | IRI PT | .27 |
| 1. Mackes et al. (2018) |  | 34 | PT | yes | Emp Acc | IRI PT | .48 |
| 1. Lewis et al. (2015) |  | 389 | PT | no | Face and Body (supramodal variable) | IRI PT | .07 |
| 1. Konrath et al. (2018) |  | 90 | PT | no | Faces | IRI PT | -.16 |
| 1. Panfilis et al. (2018) |  | 200 | PT | no | Faces | IRI PT | -.09 |
| 1. Nyline et al. (2018) | DVO | 35 | PT | no | Faces | IRI PT | -.06 |
| 1. Olderbak & Wilhelm (2017), Study 1 |  | 157 | PT | no | Faces | IRI PT | .04 |
| 1. Kim et al. (2016) | Control | 36 | PT | no | Faces | IRI PT | .05 |
| 1. Vachon & Lynam (2016) |  | 210 | PT | no | Faces | IRI PT | .05 |
| 1. Kelly (2013), study 2 ^a^ |  | 100 | PT | no | Faces | IRI PT | .12 |
| 1. Kim et al. (2016) | Narcissists | 36 | PT | no | Faces | IRI PT | .21 |
| 1. Lewis et al. (2015) |  | 389 | PT | no | Faces | IRI PT | .26 |
| 1. Nyline et al. (2018) | Controls | 35 | PT | no | Faces | IRI PT | .30 |
| 1. Brislin (2015) ^a^ |  | 66 | PT | no | Faces (avg) | IRI PT | .09 |
| 1. Besel & Yuille, (2010; also see Besel, 2007) |  | 135 | PT | no | Faces (avg) | IRI PT | .11 |
| 1. Muller et al. (2010) |  | 30 | PT | no | False Belief A | IRI PT | .08 |
| 1. Muller et al. (2010) |  | 30 | PT | no | False Belief B | IRI PT | .08 |
| 1. Muller et al. (2010) |  | 30 | PT | no | Faux Pas | IRI PT | -.14 |
| 1. Schlegel et al. (2017) |  | 131 | PT | yes | GERT | IRI PT | .39 |
| 1. Corbera et al. (2013) |  | 54 | PT | yes | Hinting task | IRI PT | -.05 |
| 1. Mar et al. (2006) |  | 94 | PT | yes | IPT | IRI PT | .05 |
| 1. Spreng et al. (2009) |  | 79 | PT | yes | IPT | IRI PT | .20 |
| 1. Carpenter et al. (2016) |  | 232 | PT | yes | IPT | IRI PT | .25 |
| 1. Dziobek et al. (2008) |  | 18 | PT | no | MET Cog | IRI PT | .28 |
| 1. Foell et al. (2018) |  | 80 | PT | no | MET Cog (avg) | IRI PT | .08 |
| 1. Muller et al. (2010) |  | 30 | PT | no | RMET | IRI PT | -.42 |
| 1. Romero-Martinez et al. (2013) | HC | 19 | PT | no | RMET | IRI PT | -.30 |
| 1. Calvi (2011) ^a^ | Men | 65 | PT | no | RMET | IRI PT | -.21 |
| 1. Spreng et al. (2009) |  | 79 | PT | no | RMET | IRI PT | -.16 |
| 1. Calvi (2011) ^a^ | Women | 159 | PT | no | RMET | IRI PT | .04 |
| 1. Gretak (2015) ^a^ |  | 185 | PT | no | RMET | IRI PT | .06 |
| 1. Lee et al. (2010) |  | 96 | PT | no | RMET | IRI PT | .06 |
| 1. Kelly (2013), study 1 ^a^ |  | 99 | PT | no | RMET | IRI PT | .08 |
| 1. Mar et al. (2006) |  | 94 | PT | no | RMET | IRI PT | .08 |
| 1. Puskar (2011) ^a^ |  | 219 | PT | no | RMET | IRI PT | .08 |
| 1. Melchers et al. (2016) |  | 742 | PT | no | RMET | IRI PT | .11 |
| 1. Melchers et al. (2015) |  | 108 | PT | no | RMET | IRI PT | .12 |
| 1. Bedwell et al. (2014) |  | 686 | PT | no | RMET | IRI PT | .14 |
| 1. Chan (2008) ^a^ |  | 238 | PT | no | RMET | IRI PT | .19 |
| 1. Lyvers et al. (2017) |  | 102 | PT | no | RMET | IRI PT | .22 |
| 1. Jankowiak-Siuda et al. (2016) |  | 175 | PT | no | RMET | IRI PT) | .30 |
| 1. Lyvers et al. (2018) |  | 161 | PT | no | RMET | IRI PT | .30 |
| 1. Vonk et al. (2013) |  | 368 | PT | no | RMET | IRI PT | .30 |
| 1. Romero-Martinez et al. (2013) | IPV | 17 | PT | no | RMET | IRI PT | .56 |
| 1. Turner (2016) ^a^ |  | 98 | PT | yes | RMFA | IRI PT | .12 |
| 1. Melchers et al. (2015) |  | 108 | PT | no | Silent Video Emotion Recognition | IRI PT | .14 |
| 1. Vonk et al. (2013) |  | 368 | PT | no | SPCRT | IRI PT | .22 |
| 1. Rogers et al. (2007) | ASD | 21 | PT | no | SST | IRI PT | .05 |
| 1. Rogers et al. (2007) | HC | 21 | PT | no | SST | IRI PT | .46 |
| 1. Puskar (2011) ^a^ |  | 219 | PT | no | SSTV | IRI PT | .15 |
| 1. Dodell-Feder et al. (2013) |  | 74 | PT | no | TOM task | IRI PT | -.07 |
| 1. Brislin (2015) ^a^ |  | 66 | PT | no | TOM task (avg) | IRI PT | -.07 |
| 1. Martinez et al. (2018) |  | 42 | PT | yes | Video Emotion Recognition | IRI PT | .36 |
| 1. Rosenblau et al. (2015) | TD | 23 | PT | yes | Video TOM Direct Mentalizing | IRI PT | .25 |
| 1. Rosenblau et al. (2015) | ASD | 28 | PT | yes | Video TOM Direct Mentalizing | IRI PT | .28 |
| 1. Rosenblau et al. (2015) | TD | 23 | PT | yes | Video TOM Indirect Mentalizing | IRI PT | -.42 |
| 1. Rosenblau et al. (2015) | ASD | 28 | PT | yes | Video TOM Indirect Mentalizing | IRI PT | .13 |
| *Note*. SR = self-report; CE = cognitive empathy; EC = empathic concern; PT = perspective-taking motivation; CON = empathic contagion; RMET = Reading the Mind in the Eyes Test; IPT = Interpersonal Perception Test; Emp Acc = empathic accuracy task; Faces = any unisensory facial emotion recognition test; TOM = theory of mind; DANVA = Diagnostic Analysis of Nonverbal Accuracy; AMT = Awkward Moments Test; BLERT = Bell-Lysaker Emotion Recognition Test; Aff = affective; Cog = cognitive; MASC = Movies for the Assessment of Social Cognition; PONS = Profile of Nonverbal Sensitivity; MERT = Multimodal Emotion Recognition Test; SST = Strange Stories Task; GERT = Geneva Emotion Recognition Test; RMFA = Reading the Mind in Films (American) test; SPCRT = Social and Physical Causal Reasoning Test; SPNV = nonverbal social perspection; SPV = verbal social perception; BES = Basic Empathy Scale; SMS = Self-Monitoring Scale; IRI = Interpersonal Reactivity Index; EQ = Empathy Quotient; ER = emotional reactivity; QMEE = Questionnaire Measure of Emotional Empathy; BEES = Balanced Emotional Empathy Scale; MEES = Mehrabian Emotional Empathy Scale ; PDA = Perceived Decoding Ability scale; BEI = Bryant Empathy Index; MRBS = Mindreading Beliefs Scale; ACME = Affective and Cognitive Measure of Empathy; AR = affective resonance; QCAE = Questionnaire of Cognitive and Affective Empathy; OS = online simulation; PROX = proximal responsivity. ^a^ = unpublished study | | | | | | | |
